# Review of Ultrasonic Particle Manipulation Techniques: Applications and Research Advances

**DOI:** 10.3390/mi14081487

**Published:** 2023-07-25

**Authors:** Shuai Wang, Xuewei Wang, Fucheng You, Han Xiao

**Affiliations:** College of Information Engineering, Beijing Institute of Graphic Communication, Beijing 102627, China; 15102552156@163.com (S.W.);

**Keywords:** ultrasonic particle manipulation, acoustic radiation force, acoustic streaming-induced force, sound field reconstruction, ultrasonic transducer arrays

## Abstract

Ultrasonic particle manipulation technique is a non-contact label-free method for manipulating micro- and nano-scale particles using ultrasound, which has obvious advantages over traditional optical, magnetic, and electrical micro-manipulation techniques; it has gained extensive attention in micro-nano manipulation in recent years. This paper introduces the basic principles and manipulation methods of ultrasonic particle manipulation techniques, provides a detailed overview of the current mainstream acoustic field generation methods, and also highlights, in particular, the applicable scenarios for different numbers and arrangements of ultrasonic transducer devices. Ultrasonic transducer arrays have been used extensively in various particle manipulation applications, and many sound field reconstruction algorithms based on ultrasonic transducer arrays have been proposed one after another. In this paper, unlike most other previous reviews on ultrasonic particle manipulation, we analyze and summarize the current reconstruction algorithms for generating sound fields based on ultrasonic transducer arrays and compare these algorithms. Finally, we explore the applications of ultrasonic particle manipulation technology in engineering and biological fields and summarize and forecast the research progress of ultrasonic particle manipulation technology. We believe that this review will provide superior guidance for ultrasonic particle manipulation methods based on the study of micro and nano operations.

## 1. Introduction

How to achieve precise manipulation of particles at the micro- and nano-scale is a popular area of research. Due to the tiny scale of nanoparticles, direct manipulation of particles by mechanical external forces is often difficult to achieve. To date, non-contact particle manipulation is usually dynamic by changing the external gradient force field, and several different forms of energy are available, including electrical [1], magnetic [2], optical [3], hydrodynamic [4], and acoustic [5] methods, which have been used to manipulate particles with high spatial resolution. The non-contact approach allows not only effective and high-precision suspension or manipulation of cells, blood, and drugs [6] but also avoids secondary contamination.

Optical actuation requires complex optical systems, including high-power lasers and high numerical aperture objectives, which may cause physiological and thermal damage to the cells [7]; magnetic actuation requires marking the target with magnetic materials, which may affect the chemical properties of the particles [8]; and electric fields require current-induced heating or direct exposure to electric fields, which may affect the particle properties [9]. Among the many techniques, non-contact manipulation of particles achieved by acoustic radiation force [10] or acoustic streaming-induced forces [11] has proven to be a promising method, and the unique advantage of acoustic fields over optical fields is that they can pass through opaque media and have significant efficiency advantages such as low power consumption and high output power. No particle pretreatment is required relative to magnetic fields (i.e., it is a label-free operation method). It is a gentle mechanical vibration method relative to electrodynamic fields, with minimal impact on cell viability and good biocompatibility [12]. Ultrasonic particle manipulation techniques show great potential for the capture and manipulation of microscopic objects.

The main component of the ultrasonic particle manipulation device is the ultrasonic transducer, which converts electrical energy into mechanical vibrations that produce acoustic waves in the fluid medium. When acoustic waves interact with the medium, two acoustic phenomena occur: acoustic streaming-induced forces and acoustic radiation forces, and one of these phenomena or a combination of them manipulates particles. Two different acoustic modes are commonly used to manipulate particles: surface acoustic wave (SAW) [13] and bulk acoustic wave (BAW) [14]. SAW is mainly generated by an interdigital transducer (IDT), where the acoustic signal propagates along the surface of a piezoelectric substrate, allowing the manipulation of tiny objects in a one- or two-dimensional plane. Depending on the generation method, SAW can be divided into two forms: traveling surface acoustic wave (TSAW) and standing surface acoustic wave (SSAW). BAW can propagate through a medium, and it is usually generated by small piezoelectric transducers [15] (PZT), which makes BAW promising for highly integrated and miniaturized [16] applications. In practice, most piezoelectric transducers are usually driven by ultrasonic frequencies [17], allowing precise manipulation of micron or even submicron particles.

In recent years, with the wide application of micro-nano technology, ultrasonic particle manipulation techniques have experienced vigorous development, and a large number of research results have been achieved in theoretical calculation methods, transducer arrangement and acoustic field design, and manipulation of various morphologies of various particles. In this paper, we present a concise review of recent research advances in operational methods, sound field generation methods, and sound field reconstruction algorithms for ultrasonic particle manipulation techniques. The characteristics of each method are described according to the sound field morphology, and we explore the effects of each method on the particle manipulation morphology and the scenarios in which they are applicable. We present the applications of ultrasonic particle manipulation techniques in the fields of engineering control, biomedicine, micro-robotics, and materials preparation. Finally, we address the limitations of current ultrasonic particle manipulation techniques, describe the current technical difficulties, and point out future research directions.

## 2. Ultrasonic Particle Manipulation Theory

There are two main acoustic forces in ultrasonic particle manipulation, namely, the acoustic radiation force and the acoustic streaming-induced forces. In order to calculate these two forces, the spatial distribution of acoustic waves in the acoustic field [18] or acoustic streaming field [19,20] must be specified.

### 2.1. Acoustic Propagation Theory

Sound propagation satisfies three fundamental laws of physics, namely, Newton’s second law, the law of conservation of mass, and the temperature, pressure, and volume relationships, from which the equations of continuity, conservation of momentum, and equation of matter for sound waves are derived. In the idealized state where the propagating medium is viscosity-free, homogeneous, stationary, and adiabatic, the three-dimensional fluctuation equation of the acoustic wave is derived as follows:

(1) $$\nabla^{2}p=\frac{1}{c_{0}^{2}}\frac{\partial^{2}p}{{\partial t}^{2}}$$

where *p*(*x*,*y*,*z*,*t*) denotes the variation of sound pressure with time, $c_{0}$ is the speed of sound in the medium, and “$\nabla^{2}$” is the Laplace operator. According to the equation of sound pressure fluctuation, the equations of sound pressure and mass velocity of a plane wave propagating along the x-axis can be obtained as follows:

(2) $$p(t,x)\;=\;p_{a}e^{j(wt-kx)}\;v(t,x)\;=\;v_{a}e^{j(wt-kx)}$$

where *k* is the wave number, $e^{iwt}$ is the time parameter, $p_{a}$ is the sound pressure amplitude, $v_{a}\;is\;the\;mass\;vibration\;velocity$. When multiple acoustic waves propagate simultaneously in the same space, the superposition interference phenomenon will occur, let the phase difference ${\;\varphi =\varphi }_{1}$ *−* $\varphi_{2}$ is a fixed value, the acoustic expression of the synthetic sound pressure P:

(3) $$P=P_{1}+P_{2}=A_{1}\cos(\mathrm{wt}-\varphi_{1})+A_{2}\cos(\mathrm{wt}-\varphi_{2})\;=A\cos(\mathrm{wt}-\varphi )$$

where the equation

(4) $$A=A_{1}^{2}+A_{2}^{2}+2A_{1}A_{2}\cos(\varphi_{2}-\varphi_{1})\;\varphi \;=\;\arctan\frac{A_{1}sin\varphi_{1}+A_{2}sin\varphi_{2}}{A_{1}cos\varphi_{1}+A_{2}cos\varphi_{2}}$$

As can be seen from the formula, sound waves have an interference effect, and the frequency of synthetic sound waves remains constant, while sound pressure amplitude and phase difference *φ* are related. Therefore, when multiple acoustic waves arrive at a location point of the same phase of vibration, the location point is of the enhanced vibration; when the phase is opposite, the location point is of the weakened vibration.

### 2.2. Acoustic Propagation Theory

The Huygens–Fresnel principle [21] can be used to calculate the sound field properties after the superposition and interference of acoustic waves in space. The method is to split the wave source, and a point *P* separated by any wavefront surface *S*. The resulting surface element *dS* is regarded as the new wave source, and the sound pressure at the field point *P* is obtained by superimposed interference of each surface element *dS*. Assuming that the wavefront surface *S* is planar (*z* = 0), the sound pressure *P*(*r*) at a site in space can be expressed as

(5) $$P(r)\;=\;\frac{i}{\lambda }\iint p_{0}\left(r_{0}\right)\frac{e^{-ikr}}{r}\cos\theta dS$$

where *r* is the distance from the surface element *dS* to the field point *P,* θ is the azimuthal angle of the field point *P* with respect to the surface element *dS*. In addition to the Huygens–Fresnel principle, the far-field characteristics of the piston model [22] can be used for sound pressure calculation, such as taking the piston plane as the *xy* plane and the total acoustic radiation pressure *P* at any point *r* in space is linearly superimposed by the acoustic radiation pressure of each transducer as follows:

(6) $$P(r)=\sum_{n=1}^{N}P_{n}A\frac{D_{f}(\theta_{n})}{d_{n}}e^{i(\varphi_{n}+kd_{n})}$$

where *N* represents the number of array elements in the transducer array, $P_{n}$ is the constant defined by the transducer power, *A* is the peak amplitude of the excitation signal, $d_{n}$ is the distance from the center of the transducer to point *r*, $\varphi_{n}$ is the phase of the transducer, *k* represents the wave number, and $D_{f}$ represents the far-field directivity function:

(7) $$D_{f}\;=\;2J_{1}(\mathrm{kasin}\theta )/(\mathrm{kasin}\theta )$$

where $J_{1}$ represents the first-order Cybel function, *a* is the radius of the transducer, and *θ* is the angle between the point *r* and the *z*-axis.

### 2.3. Acoustic Radiation Force and Acoustic Streaming-Induced Forces

Ultrasonic propagation encounters particles (size much smaller than the wavelength) that will be reflected, scattered, refracted, and have other physical reactions. Gor’kov’s theoretical model [23] assumes that acoustic waves exchange momentum with particles to generate forces. When there is a gradient sound field in space, the object is subjected to acoustic radiation forces depending mainly on the acoustic gradient force, and the value of the acoustic gradient *U* and the acoustic radiation force *F* are equal in magnitude and opposite in direction:

(8) $$U=2\pi R^{3}\left[\frac{\varphi_{1}}{3\rho_{0}c_{0}^{2}}{\left(p_{1}^{in}\right)}^{2}-\frac{\varphi_{2}\rho_{0}}{2}{\left(u_{1}^{in}\right)}^{2}\right]\;\varphi_{1}=1-\frac{\rho_{0}c_{0}^{2}}{\rho_{p}c_{p}^{2}},\;\varphi_{2}=2\frac{\rho_{p}-\rho_{0}}{\rho_{0}+2\rho_{p}}$$

(9) F=−∇U

where *R* is the spherical target radius, $\rho_{0}$ is the medium density, $\rho_{p}$ is the target object density, $c_{0}$ is the speed of sound in the medium, $c_{p}$ is the speed of sound in the target object, $p_{1}^{in}$ is the first-order average incident sound pressure, $u_{1}^{in}$ is the average particle vibration velocity, ∇ is the gradient operator, and $\varphi_{1}$ and $\varphi_{2}$ denote the scattering coefficients of the resonance model.

In most cases, the acoustic radiation force is the main driving force for particle manipulation. However, in some specific cases, the state of motion of the particles is the result of the combined action of two forces, the acoustic radiation force, and the acoustic streaming-induced forces. For a uniformly isotropic fluid, the acoustic streaming-induced forces can be determined from Stokes’ law [24]:

(10) $$F_{drag}=6\pi \mu R(\bar{v_{2}}-v_{p})$$

where the kinetic viscosity of water is μ = Pa·s, *R* is the particle size, $v_{p}$ is the velocity of the particle, and *(*$\bar{v_{2}}$ *−* $v_{p}$*)* represents the velocity of the particle relative to the liquid, and the motion of the particle is governed by Newton’s second law:

(11) $$\frac{d}{dt}\left(m_{p}v\right)=F_{rad}+F_{drag}$$

where $m_{p}$ is the particle mass and the acoustic radiation force $F_{rad}$ is determined by the Gorkov equation.

## 3. Ultrasonic Particle Manipulation Mechanism

Currently, acoustic radiation force and acoustic streaming-induced forces are the two most widely used physical effects in ultrasonic particle manipulation. Particles (nanometer to micrometer scale) can be manipulated using either combination of their effects [25]. Depending on the manipulation mechanism, they can be generally classified into three categories: standing waves, traveling waves, and acoustic streaming.

### 3.1. Acoustic Radiation Manipulation

#### 3.1.1. Standing Wave Manipulation Particles

**Figure 1:**
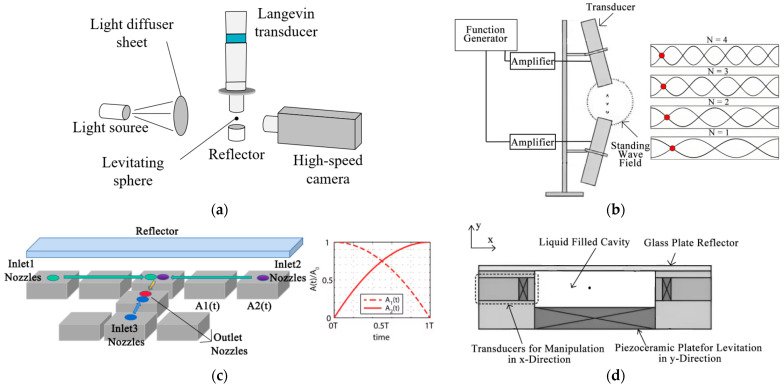
Schematic diagram of the particle manipulation device. (**a**) Non-contact manipulation of standing wave levitation by adjusting the moving wall displacement. (**b**) Switching the transducer between different frequency modes to manipulate the object. The figure on the right shows the captured nodes of the particles at different frequencies of the acoustic wave. Reprinted with permission from [30]. (**c**) Controlling the amplitude of adjacent transducers to manipulate the movement and mixing of droplets, which are eventually delivered to the outlet. The figure on the right shows the amplitude variation of two adjacent transducers. Reprinted with permission from [31]. (**d**) The device manipulates the object by the phase difference between two opposing transducers in a pendant-filled liquid cavity. Reprinted with permission from [32].

Ultrasonic particle manipulation based on the standing wave principle has been widely used in the study of aeroacoustic levitation [26], cell manipulation [27], and the alignment of nanomaterials [28]. There are various methods to manipulate particles using standing waves, such as changing the distance position of the resonant cavity in the acoustic device; the study by Marco A. B. Andra et al. [29] used a uniaxial acoustic levitator with a movable reflector, and they found the optimal capture position of the sphere by controlling the resonant cavity distance, i.e., the position under which the sphere has the best levitation stability and the optimal oscillation behavior. On this basis, the frequency and amplitude of the harmonic signal of the ultrasonic probe were controlled to study the oscillatory behavior of the levitated sphere, as shown in Figure 1a; this method requires strict accuracy of the acoustic device group, which is difficult to meet in practice. The simpler and more efficient method for standing wave manipulation is to change the transducer operating parameters (e.g., frequency, phase, amplitude). Teruyuki Kozuka et al. [30] generated a standing wave field by two tilted transducers without using a reflector and achieved constant velocity transport of particles by changing the transducer frequency and electrode switching, as shown in Figure 1b. Daniele Foresti et al. [31] used a system consisting of eight ultrasonic probes and a reflection device that periodically modulates the amplitude of adjacent transducers, allowing the operation of complex transport and suspension mixing of water droplets with a radius of 0.84 mm, as shown in Figure 1c. All of the above studies were performed in an air medium, and there are also attempts to achieve ultrasonic particle manipulation in water, such as the experimental study by Courtney, C. R. P et al. [32] to control the phase of two transducers to achieve complex motion of small spheres with a diameter of 5 µm in water, as shown in Figure 1d. Among the many control methods, phase control requires only a simple phase delay of the transducer, and the acoustic wave can be focused at a point in space, so the phase control method has gradually become the main development trend of standing wave ultrasonic manipulation technology [33].

Standing waves can generate significant acoustic radiation forces to capture specific particles between acoustic devices, but standing wave sound fields have limited application scenarios because they require at least one set of acoustic devices with strict relative positions [34], and in addition, are not suitable for single tiny objects and complex manipulations. Ultrasonic particle manipulation based on single or multiple transducers generating traveling waves or focused acoustic fields can avoid these problems.

#### 3.1.2. Traveling Wave and Focused Sound Field Manipulation Particles

**Figure 2:**
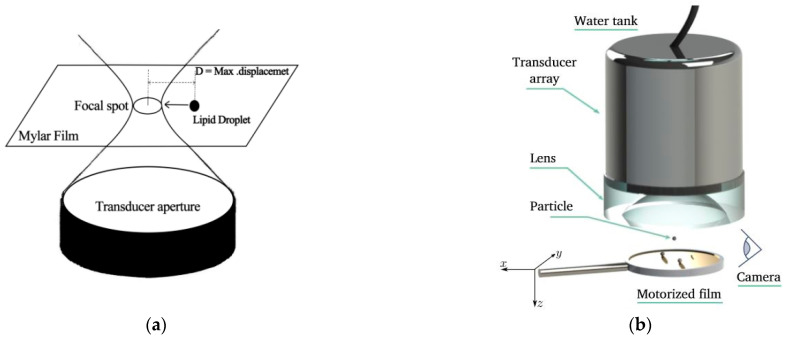

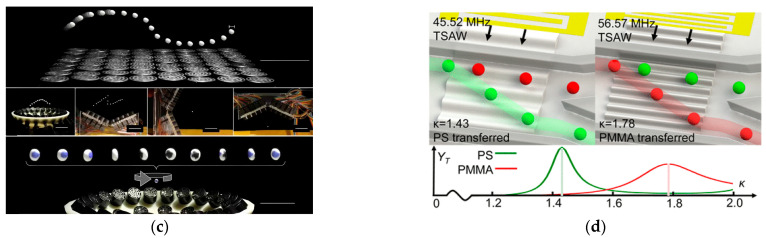
Schematic diagram of the particle manipulation device. (**a**) Single-Focused Acoustic Beam Acoustic Tweezer (SBAT) to control the particles on the axis. (**b**) The ultrasonic transmitter focuses the acoustic beam to keep the polystyrene particles in a stable suspension in three dimensions. Reprinted with permission from [40]. (**c**) Capture, pan, and rotate particles using different arrangements of arrays without moving the array. Reprinted with permission from [41]. (**d**) TSAW screening separates polystyrene (PS) and plexiglass (PMMA) microparticles. Reprinted with permission from [42].

Ultrasonic particle manipulation based on the traveling wave principle has been widely used for cell mechanics measurements [35,36] and spatial manipulation of living cells and microorganisms [37,38]. Traveling wave manipulation can be achieved over a larger spatial area without the need to surround the target with acoustic emission elements. There are several ways to manipulate particles using traveling waves, such as using a single-array transducer to form an aggregated acoustic beam. Jungwoo Lee et al. [39] at the University of Southern California used a single-array focused transducer with a center frequency of 30 MHz to pull the particles back on the axis, as shown in Figure 2a. Baresch et al. [40] used a single-array transducer to form an acoustic beam to achieve non-contact push–pull manipulation of positive-contrast particles and also to precisely control the position of the particles and the magnitude of the force on them, as shown in Figure 2b. This study provides an important theoretical and experimental basis for single-array transducers to manipulate elastic microparticles in three-dimensional space. Compared to a single-array transducer with a single mode of operation, modulating the emitted acoustic waves (e.g., frequency, amplitude, phase) of each array of ultrasonic transducers allows for more flexible manipulation. Marzo A et al. [41] used multiple planar ultrasonic transducer array groups to create a continuous, dynamically adjustable BAW acoustic field in the air with a range of morphology and focus positions, enabling versatile manipulation of particles, as shown in Figure 2c. In addition to particle manipulation using traveling waves in air media, traveling waves can also be used to achieve screening of particles with different properties within a microfluidic cavity. Ma Z et al. [42] studied the resonance characteristics of particles in traveling wave fields, and they used an interdigital transducer of different frequencies to precisely screen particles of different materials within a microfluidic cavity, as shown in Figure 2d. These research works on acoustic manipulation techniques have provided a variety of methods for research in ultrasonic particle manipulation.

The gradient of the acoustic field generated by traveling waves is weak, and the particles are subject to smaller acoustic radiation force, vulnerable to external interference, and less stable, but the equipment used for traveling wave manipulation is simpler compared to standing waves and more suitable for modulating any pair of patterns or individual objects in real time.

### 3.2. Acoustic Streaming-Induced Forces Manipulation

**Figure 3:**
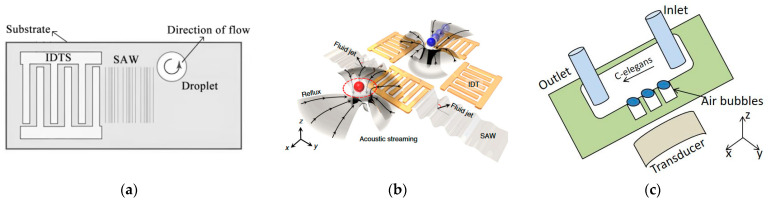
Schematic diagram of the acoustic streaming-induced forces manipulation particle device. (**a**) The droplets affected by the asymmetric SAW distribution produce a fast acoustic streaming; the arrows represent the horizontal projection of the acoustic streaming. Reprinted with permission from [46]. (**b**) Three-dimensional acoustic streaming-induced hydrodynamic trap device consisting of four IDTs in one small unit for fluid transport. Reprinted with permission from [47]. (**c**) Diagram of the device in which a piezoelectric transducer generates acoustic waves to drive air microbubbles in a microcavity.

The fluid medium in an acoustic field gains momentum by absorbing acoustic waves, and when the acoustic streaming effect plays a major role, the particles move with the fluid motion. Ultrasonic particle manipulation based on the acoustic streaming principle has been used for submicron particle trapping [43], three-dimensional manipulation combined with acoustic radiation forces [44], and manipulation of biological single cells and tissues [45]. Acoustic streaming-induced forces manipulation is the indirect manipulation of particles by acoustic waves inducing a steady flow of the medium fluid or by using the physical properties of the particles to selectively manipulate them within a microfluidic cavity. Different particle sizes in the acoustic streaming field are subjected to different tug forces. Rogers et al. [46] achieved particle separation by creating a cyclonic flow within the droplet, where particles of different sizes are subjected to different radiation and acoustic streaming-induced forces, as shown in Figure 3a. Acoustic streaming-induced forces manipulation can not only generate sufficient local acoustic radiation force to separate solid particles in fluid media but also to rotate individual particles, cells, and organisms. For example, Steven Peiran Zhang et al. [47], based on an interdigital transducer using a combination of acoustic surface waves and acoustic streaming, achieved the manipulation of alignment, translation, and rotation of tiny particles, cells, droplets, etc., in a microfluidic cavity channel, as shown in Figure 3b. Precise rotational manipulation of individual cells or organisms is very important in biological applications, and previous manipulation techniques often make it difficult to precisely manipulate large organisms. However, Ahmed et al. [48] used a device that immobilizes microbubbles within the sidewall microcavities of microchannels, and they achieved precise rotation of colloidal particles, cells, and whole organisms (e.g., Cryptobacterium histolytica) by pushing the microbubbles to generate oscillatory motion and thus stable microvortices, as shown in Figure 3c.

Acoustic streaming-induced forces manipulation effectively overcomes the disadvantages of low flexibility and single mode of manipulation of acoustic radiation force manipulation and expands the scope of use of acoustic manipulation techniques. The characteristic of the acoustic streaming-induced forces manipulation technique is the low temperature of the control zone, which has a greater advantage in handling temperature-sensitive samples [49]. It is expected that the acoustic streaming-induced forces manipulation method can be used to automate the separation of submicron particles and instantaneous analysis equipment by effectively designing and adjusting the applied acoustic field.

## 4. Ultrasonic Particle Manipulation Method

### 4.1. Particle Manipulation Based on Ultrasonic Transducer Arrays

**Figure 4:**
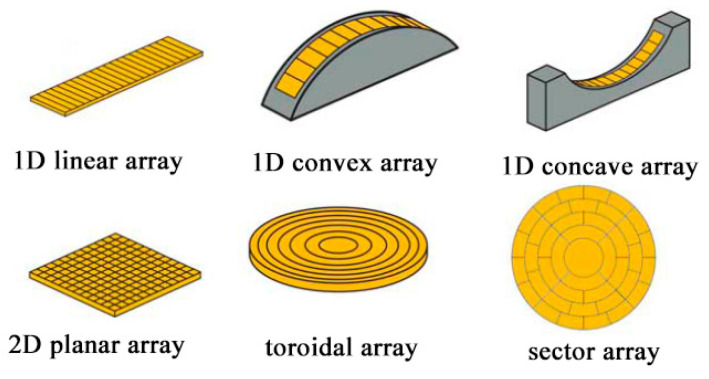
Schematic layout of the ultrasonic transducer array.

The shape of the sound field generated by a single array or two transducers is generally fixed, and the particles are subjected to a fixed acoustic radiation force in the sound field, which makes it difficult to manipulate them flexibly and multi-functionally. With the development of transducer processing and circuit-driven systems, designs with combined arrangements of multiple transducers have emerged [50], where the emission signal (frequency, phase, intensity, duration, etc.) of each transducer in an ultrasonic transducer array can be controlled independently. The ultrasonic transducer array form can generate a modulated acoustic field without the need for physical lenses, custom transducers, or mechanical drives, and this method is currently the best solution for providing dynamic particle manipulation. Ultrasonic transducer arrays can be divided into 1D linear arrays, convex or concave arrays, toroidal arrays, sector arrays, 2D planar arrays, and various other ways according to the arrangement of the array elements, as shown in Figure 4.

**Figure 5:**
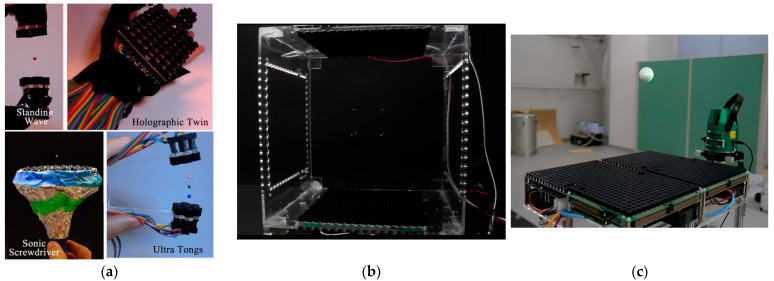
Schematic diagram of the transducer array-based particle manipulation device. (**a**) Wearable acoustic particle manipulation device. Reprinted with permission from [51]. (**b**) Dynamic modulation of acoustic field based on multiple transducers for object manipulation in three-dimensional space. Reprinted with permission from [52]. (**c**) Acoustic levitation manipulation of 30 mm polystyrene spheres. Reprinted with permission from [53].

The acoustic emission power and detection sensitivity of ultrasonic transducer arrays are far superior to those of single array elements. Its greatest feature is that it can control the acoustic beam morphology flexibly and effectively, which enriches the variety of particle motion and manipulation methods and greatly expands the application scenarios of ultrasonic particle manipulation. Asier Marzo et al. [51] proposed a wearable miniaturized particle manipulation device that allows the capture, movement, transfer, and combination of individual particles by hand, as shown in Figure 5a; the wearer can achieve dexterous and complex non-contact manipulation by hand, but the number and power of transducer elements are small, resulting in limited manipulation capability. To achieve finer control, ultrasonic transducer arrays require complex arrangements and more devices; Marzo et al. [52] used a phased transducer array with 256 array elements, as shown in Figure 5b, and this device adjusted the excitation signal to achieve a wide range of acoustic beam deflection without moving the transducer. The precise superposition of interfering acoustic waves from the transducers produces a variety of complex acoustic field patterns, enabling a variety of flexible operations such as simultaneously and independently capturing, moving, and rotating any multiple particles in the air. While most studies tend to stop at tiny objects of light weight due to the limited power emitted by transducers, Seki Inoue et al. [53] at the University of Tokyo constructed a holographic acoustic field to manipulate a 30 mm ultra-large polystyrene sphere by designing an array of 996 transducers, as shown in Figure 5c. This large device provides experimental guidance for acoustic levitation to manipulate large-diameter objects.

**Figure 6:**
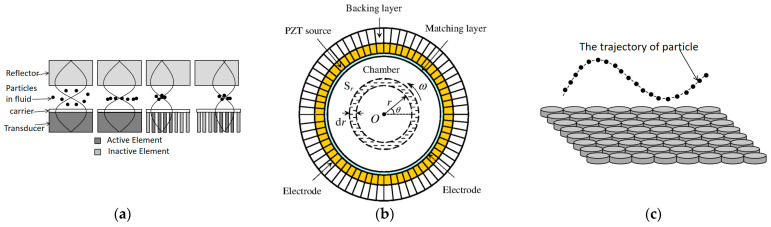
Schematic diagram of the transducer array-based particle manipulation device. (**a**) One-dimensional array of suspended particles. (**b**) The 64-array annular transducer device generates an acoustic vortex field, with the gray dashed coil representing the direction of rotation. Reprinted with permission from [55]. (**c**) The particles are translated, rotated, and manipulated using different arrangements of single-sided ultrasonic transducer arrays.

Different arrangements of ultrasonic transducer arrays are used for different purposes. The transducer circuit of one-dimensional linear arrangement is simple to build and is often used for simple levitation and translation of particles. Peter Glynne-Jones et al. [54] formed a standing wave between the one-dimensional ultrasonic transducer array and the reflector plate to achieve particle levitation and movement, as shown in Figure 6a. The concave or ring-shaped arrangement of transducers can achieve a better formation of a vortex acoustic field in the medium space, which drives the particle rotation by acoustic radiation moment. Current research can appear as vortex sound fields in various media. For example, Hong et al. [55] used a 64-array annular transducer to generate a two-dimensional Bessel vortex sound field in a microcavity channel, as shown in Figure 6b. Acoustic radiation torque is generated on the surface of the particle under the action of the acoustic field gradient, the magnitude of which depends on the strength of the vortex acoustic field and the acoustic impedance of the particle [56], and the orbital angular momentum in the vortex acoustic field is transferred to the particle to realize the rotational manipulation of a fluid mixture composed of particles and water. In addition, Marzo et al. [57] used 192 transducers to form an annular phased array with an open spherical sector to achieve the capture of particles with wavelengths comparable to acoustic waves in an air medium. Ultrasonic transducer arrays in two-dimensional planes are more amenable to three-dimensional manipulation of particles. Marzo et al. [41] utilized multiple sets of two-dimensional transducer arrays in different arrangements to capture, translate, and rotate multiple particles simultaneously, as shown in Figure 6c, experimentally demonstrating the flexibility of planar ultrasound transducer arrays for particle manipulation and expanding the application scenario.

Ultrasonic transducer arrays can generate arbitrary patterns of sound fields to accomplish flexible, dynamic manipulation, which provides fundamental support for various scenario applications. However, ultrasonic transducer arrays require the construction of programmable multichannel drive systems and precise mathematical manipulation models for the independent control of each transducer circuit. For example, Antoine Riaud et al. [58] developed a transducer array consisting of 32 IDTs with programmable electronics and applied inverse filtering techniques to synthesize surface acoustic waves of a specific wavefield. This system allows not only three-dimensional manipulation of single or multiple cells, such as displacement and rotation, but also generates vortex-type surface acoustic waves for precise control of vortices, as well as for precise manipulation and handling of stationary droplets. This work has important implications for microfluidics and biomedical applications.

However, the complexity and cost of transducer arrays become higher as the flexibility of particle manipulation and the weight of particles increase, and these difficulties make the design and fabrication of ultrasonic transducer arrays difficult. To address these difficulties, some researchers would simplify the system design by techniques such as sparsely arranged transducer arrays [59,60] and element multiplexing [61]. For example, Hu Qi et al. [62] developed a three-dimensional acoustic tweezer consisting of a two-dimensional phased array to generate multiple acoustic traps, and the different traps were time-multiplexed to form an integrated acoustic field between them to achieve three-dimensional levitation and translation of positive acoustic contrast particles in water. This time multiplexing approach has the advantage of reducing the number of components and increasing the flexibility of the system, but it also introduces time delays and increases the computational and control complexity, which further limits the application of the technology. In specific application scenarios, the sound field pattern is almost fixed, and there is no need to independently modulate the excitation signal of each transducer. Therefore, the low-cost design of some application-specific sound field morphologies is also a research direction for the application of ultrasonic particle manipulation technology.

### 4.2. Particle Manipulation Based on Structural Sound Field

Particle manipulation based on structural sound fields can be applied to static sound fields for specific driving purposes. Artificially designed structural parameters and material parameters can enable acoustic materials to achieve a variety of acoustic properties [63], and the planar acoustic waves from the transducer are transmitted or reflected by such artificially designed acoustic structural devices, causing the original acoustic signal to change and generating a local acoustic field to achieve acoustic radiation force manipulation. Structural materials can be applied to ultrasonic transducers to generate specific acoustic field models, which not only simplifies the design of acoustic devices but also requires low fabrication costs and electronic systems. This provides a new approach to ultrasonic particle manipulation techniques.

**Figure 7:**
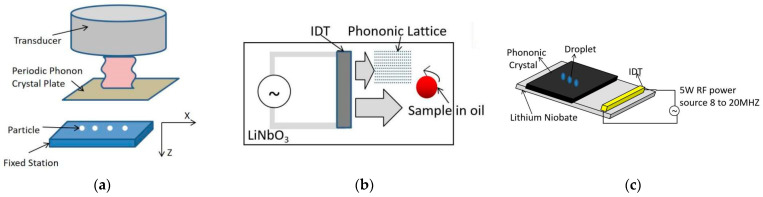
Schematic diagram of particle manipulation device based on structural sound field. (**a**) The phonon crystal plate generates a localized acoustic field to manipulate and screen particles. (**b**) Schematic diagram of a phonon crystal plate forming an asymmetric acoustic field to achieve capture of cells within a droplet. (**c**) Schematic diagram of the device and the phonon crystal plate to realize the droplet injection effect.

The interaction between acoustic waves and matter has enriched the means of acoustic wave modulation, and many novel acoustic wave propagation effects have been realized, such as the resonance [64], transmission [65], reflection, and refraction effects [66] of acoustic waves and phononic crystals to form acoustic field patterns that can be used for acoustic manipulation. Li Fei et al. [67] used the resonance effect between phononic crystal plates and acoustic waves to generate a local acoustic field for particle capture, alignment, movement, and screening, as shown in Figure 7a; this method is based on the special acoustic properties of phononic crystals, which can generate a stable, broadband and flexible tunable acoustic field. Artificially designed phononic crystals with energy band or forbidden band properties can obtain a variety of acoustic field patterns. Rab Wilson et al. [68] placed phononic crystals on the IDT propagation path to block the propagation of SAW in a certain frequency band and finally obtained an asymmetric acoustic field to achieve the aggregation of cells inside the droplet, as shown in Figure 7b. In addition, Cooper et al. also obtained a locally enhanced acoustic field to achieve the droplet injection effect by artificially modulating the geometric parameters of the phonon crystal [69], as shown in Figure 7c. Phononic crystal-based manipulation devices are easy to process and have the advantage of being disassembled and reused repeatedly to modulate SAW as partial components coupled to a microfluidic chip. This provides a structurally simple and less costly design approach for acoustic structure-based particle manipulation.

**Figure 8:**
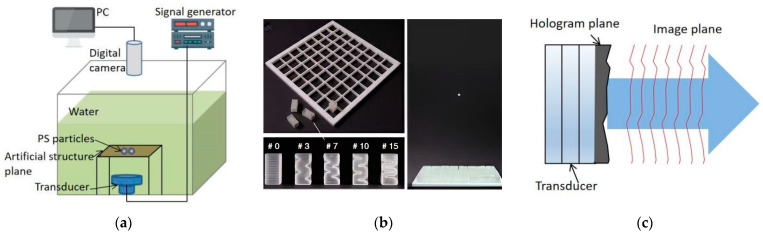
Schematic diagram of particle manipulation device based on structural sound field. (**a**) Spin particles based on surface spiral structure induced vortex field. (**b**) On the left side of the image is a photograph of the fabricated bricks and the grid holding them, and on the right side of the image is an acoustic suspension of polystyrene beads using a 4-bit focusing element surface. Reprinted with permission from [72]. (**c**) Holographic acoustic lens-based induced arbitrary acoustic field moving and rotating particles.

Specific acoustic structures can generate corresponding acoustic fields such as vortex fields, focusing fields, or arbitrary morphological acoustic fields to achieve manipulation of objects. Wang T et al. [70] obtained a Bessel vortex field by etching an Archimedean spiral structure on a copper plate, which can induce the vortex motion of particles, as shown in Figure 8a. Later, Wang T et al. [71] used the same principle to fabricate an acoustic vortex lens using a silicon substrate and successfully manipulated the rotation of shrimp eggs in the acoustic field. The design of the surface spiral structure simplifies the acoustic equipment with simple and low-cost technology, but there are difficulties in adjusting the acoustic field parameters and the single acoustic field mode. Memoli et al. [72] used acoustic metamaterials to modulate the phase of the acoustic waves to obtain a spatially focused acoustic field, which can achieve focused suspension of airborne particles, as shown in Figure 8b. The acoustic metamaterial modules can be replaced and assembled to form an arbitrary sound field, solving the problem of a single acoustic field pattern, but the formed sound field cannot be dynamically reconfigured. Kai Melde et al. [73] used 3D-printed holographic acoustic lenses to form complex acoustic fields of arbitrary shape on a flat surface, enabling selective manipulation of particles, as shown in Figure 8c. High-resolution holographic acoustic lenses can encode multiple images at different frequencies with much less computational complexity and fabrication cost than conventional transducer arrays, and the formed sound fields can be dynamically varied over a limited range.

Structural sound field-based methods for manipulating particles enrich the acoustic wave form, and the sound field has the advantages of high information content, strong pre-programming capabilities, and simple reproduction, but the sound field is static and not suitable for dynamic applications, nor can it be dynamically reconfigured like tweezers [52]. The structural-acoustic field can greatly regulate the intensity, range, and mode of action of the acoustic field with the particles, which can obtain multi-form, wide-range, and high-precision manipulation means and is expected to enhance and improve the acoustic manipulation capability, which has positive significance in the direction of miniaturization and integration in the field of acoustic manipulation.

## 5. Application of Ultrasonic Particle Manipulation

The accuracy of the sound field reconstruction is an important factor in determining the performance of the system and a key core in determining the effectiveness of sound field manipulation. A low-quality sound field may lead to inaccurate suspension capture points and affect the accuracy of particle manipulation. For ultrasonic particle manipulation systems based on transducer arrays, solving the phase distribution of the corresponding transducer array backward from the acoustic field distribution is a mathematically difficult nonlinear system to solve. An effective method is still lacking, so the exact reproduction of the phase distribution by the expected acoustic field is a hot topic in acoustic field research. There are several methods in the existing literature to solve this problem. The traditional method approach [52] is solved by optimization; the computational cost of this method is exponentially related to the size of the transducer array, and in practice, the real-time sound field reconstruction is crucial for the stability and controllability of the manipulation, which makes it difficult to apply the technology based on the traditional method to complex and variable fields. Emerging methods include machine learning-based methods, which have unmatched real-time performance and high reconstruction accuracy of the holographic sound field. The optimization methods and optimization methods of sound field reconstruction algorithms are shown in Table 1.

### 5.1. IB Algorithm and IASA Algorithm

**Figure 9:**
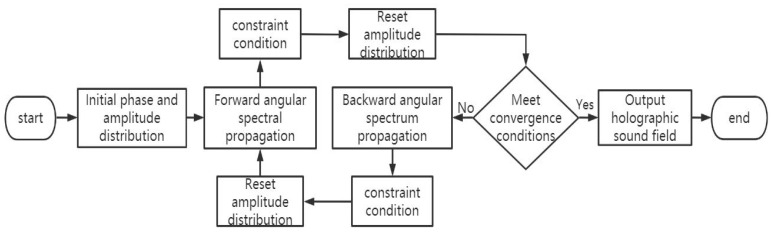
Holographic acoustic field reconstruction process based on automatic differentiation.

In terms of optimization methods for acoustic metamaterials, Melde et al. [73] proposed the Iterative Angular Spectrum Algorithm (IASA) in 2016, which is an adaptation of the GS algorithm [74] applied to acoustic holograms and can be used to calculate the phase map distribution problem in acoustic holographic acoustic lenses. By using the target image amplitude as the input of IASA, Melde et al. [73] calculate the phase distribution of the target image at 2.06 MHz ultrasound, then 3D print a transmission hologram containing the phase information, and place this acoustic hologram on the ultrasound propagation path, which can reconstruct a high-resolution ultrasound image. The IASA algorithm is suitable for solving the reconstruction of the two-dimensional holographic sound field, but the algorithm does not normalize. The flow chart of the IASA algorithm is shown in Figure 9.

In terms of optimization methods for PAT controllers, Asier Marzo et al. [52] proposed the iterative backpropagation algorithm (IBP) in 2018; it is a modified algorithm from the IASA algorithm applied to PTA that can be used to calculate the phase distribution problem of transducer arrays in 3D holographic sound fields. Marzo et al. [52] used the IB algorithm to generate focal points to manipulate multiple particles and dynamically control the emitter phase to move the focal point and, thus, the particles. In addition, the IB algorithm can generate multiple binary traps at arbitrary positions and in different directions, enabling functional holographic acoustic tweezers (HAT). However, the IB algorithm cannot be mathematically proven to converge, and the iterative approach is time-consuming and difficult to apply in real time, and its performance decreases dramatically once the holographic acoustic field becomes complex.

### 5.2. GS-PAT Algorithm

The GS algorithm can be used to recover the extracted phase information from the intensity information of a known signal, but many limitations of the traditional GS algorithm make it inappropriate for PTA devices, such as the non-planar arrangement of PTAs and arbitrary 3D positions. In order to be able to compute multipoint sound fields using PAT at high rates, in 2020, Diego Martinez Plasencia et al. [75] reformulated the GS algorithm to reduce the complexity of the GS algorithm and proposed a phase recovery algorithm, the GS-PTA algorithm, which can be used to solve high-speed multipoint phase and amplitude optimization problems of PAT devices in acoustic fields, and experimentally demonstrated that the algorithm can reconstruct acoustic fields with multiple focal points or traps at very high interaction rates. The GS-PAT algorithm can also be applied to control acoustic fields from single to multiple high-speed points with strong generality.

Diego Martinez Plasencia et al. [75] compared the GS-PTA algorithm with advanced algorithms used in multipoint levitation and haptic feedback and showed that the GS-PTA algorithm could generate high-quality ultrasonic multipoint levitation sound fields at a higher computational rate with improved accuracy and computational performance compared to the IBP algorithm, which is limited to phase modulation. The GS-PTA and Eigensolver algorithm [76], which modulates both amplitude and phase, can achieve the ability to generate ultrasonic haptic feedback points at a higher computational rate while retaining the capability of the multipoint algorithm and can reconstruct a higher quality multipoint acoustic field.

### 5.3. GS-PAT Algorithm

The least squares method (LSM) is a mathematical optimization method to solve fitting problems, which can be described as an algebraic process of fitting data using a system of linear equations. However, LSM is not satisfactory for complex problems, especially nonlinear problems. After optimization improvements by Kenneth Levenberg et al. [77], an iterative algorithm for solving nonlinear fitting problems, the Levenberg–Marquardt algorithm [78] (LMA), was proposed. The algorithm is computed by first giving an initial value and then iterating several times to determine reasonable values of the parameters in the constructed equations.

The LMA algorithm can be used for phase optimization of transducers in PTA devices, and Emiri Sakiyama et al. in 2020 [79] proposed an accurate semi-airborne haptic reproduction system that maintains the constraint of equal transducer amplitude and introduced the LMA algorithm to determine the drive signal of the transducer on the airborne ultrasonic haptic display (AUTD), which was tested in real and simulated environments for controls. The LMA algorithm, like the IBP algorithm, can only optimize the phase of the transducer, and the LMA algorithm can only find a local minimum, not necessarily a global minimum, which may seriously distort the reproduced pressure distribution as the optimization problem to be solved in the holographic sound field becomes complex.

### 5.4. Automatic Differentiation-Based Methods

In general, optimization methods that control both amplitude and phase can reconstruct higher-quality holographic acoustic fields than pure phase methods. However, Tatsuki Fushimi et al. [80] proposed a pure phase gradient descent algorithm with automatic differentiation [81], Diff-PAT, in 2020, which was experimentally shown to have higher reconstruction accuracy compared to conventional algorithms when only phase control is used and achieved performance enhancement by simply applying the DIFF-PTA algorithm to the optimization process of acoustic holograms to obtain high-quality acoustic holograms without changing the basic design of Melde [73]. The improved acoustic holograms can also be applied to the application of PTAs without any changes to the control system; DIFF-PTA is more accurate than the traditional GS-PAT algorithm and can obtain higher accuracy in sound field reconstruction while allowing the PAT controller to remain simple in design. For PATS with a large number of transducers (M = 1024), the DIFF-PAT method outperforms the Eigensolver method both in terms of accuracy and computational efficiency.

Neural networks have powerful fitting abilities to accomplish complex linear or nonlinear mappings, and Chengxi Zhong et al. [82] used a deep learning-based method, AcousNet, in 2021 to solve the inverse mapping problem from sound field distribution to phase distribution by training to allow the VGG-based neural network model to learn a large number of inverse mapping relations to directly predict the phase distribution of the PTA, which was experimentally demonstrated to be well reconstructed and usable for practical applications, with significantly shorter computation time and faster reconstruction compared to traditional optimization algorithms (IBP) and competitive prediction accuracy and real-time performance in PTA-based holographic sound field generation. It is expected that this acoustic holographic optimization method will improve the performance of many applications by reconstructing high-quality holographic acoustic fields in real time.

## 6. Application of Ultrasonic Particle Manipulation

Ultrasonic particle manipulation technology has the advantages of no damage, no contact, good biocompatibility, no additional biochemical labeling of particles, etc. These advantages will make ultrasonic particle manipulation more useful in biomedicine, materials science, engineering control, micro-robotics, and other fields, with a wide range of application prospects and promoting new applications of ultrasonic energy transfer.

### 6.1. Application in Engineering Control

In the field of precision electronics manufacturing and assembly, processing and transporting parts need to be more precise, and surface damage or structural damage can cause failure in product production, resulting in a great waste of resources in human and material resources. Acoustic manipulation techniques can solve the problem of contactless pickup and release of microcomponents. Kun Jia et al. [83] proposed a method for contactless pickup of particles from rigid surfaces using acoustic radiation forces. They achieved the pickup and localization of silica and aluminum particles (between 100 μm and 500 μm) using an experimental setup that can vary the size and position of the acoustic source. Acoustic manipulation provides new ideas for precision transport. Jianxin Meng et al. [84] proposed a method to capture microparticles from non-customized rigid surfaces and manipulate them freely. During long-distance transport, microparticles are stably suspended in liquid, thus protecting fragile and easily contaminated targets, and this method provides a promising tool for microstructure or cell manipulation. With the advantages of high control accuracy, cost reduction, practical operation time, and waste reduction, acoustic manipulation-based methods are receiving increasing attention in the field of precision electronics as they avoid damage to the device during manipulation. It is expected that chip non-contact manipulation and soldering technology based on ultrasonic particle manipulation technology will become a hot research topic in electronic assembly. It can be expected that ultrasonic particle manipulation technology will become a popular research topic in the field of electronic assembly, such as chip non-contact manipulation and soldering technology.

### 6.2. Applications in Biomedicine

The manipulation of biomolecular nanoparticles (e.g., proteins and lipids) using ultrasound techniques has attracted much attention in medical applications. For example, Filip Petersson et al. [85] proposed a method to separate lipid particles from blood using ultrasound standing waves. They introduced a half-wavelength standing wave field in the channel to move red blood cells and lipid particles in different directions, thus achieving efficient removal of lipid particles while retaining most of the red blood cells. Shuting Pan et al. [86] proposed a method to remove nonspecific binding (NSB) and detect proteins. They achieved vortex-induced removal of NSB proteins by generating multiple micro-vortices through an ultrasound resonator.

**Figure 10:**
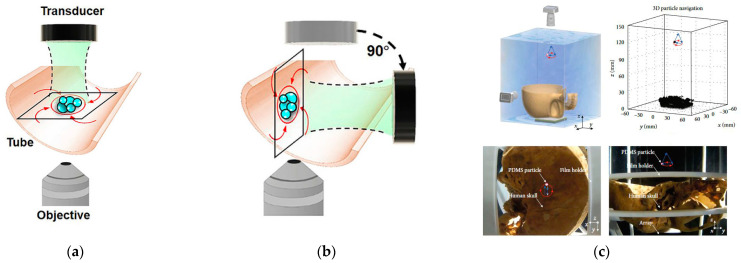
Acoustic vortexes observed under microscope to achieve drug residence. (**a**) Under static conditions, the transducer was placed on top to capture for top-down observation. Reprinted with permission from [89]. (**b**) The transducer was rotated by 90° and observed sideways. Reprinted with permission from [89]. (**c**) Schematic diagram of time-reversing acoustic tweezers through a living human skull. Reprinted with permission from [90].

Acoustic waves can penetrate complex media in living organisms at low incident power. Kirk Shung’s team at the University of Southern California has successfully trapped particles in vascular mimics using single-focused acoustic beam acoustic tweezers (SBAT)—a study that provides an exploration of the feasibility of in vivo applications of acoustic tweezers in biomedicine [87]. Recent studies have found that acoustic tweezers are moving toward in vivo applications, where high blood flow velocities in the human body make it difficult for biotherapeutics to lodge and accumulate in the circulatory system, which limits drug concentrations in the targeted region. Diego Baresch et al. [88] controlled the remote localization and activation of drug-laden particles in vivo for targeted drug delivery by using vortex beam-based acoustic traps. Wei-Chen Lo et al. [89] proposed a vortex tweezer that uses an acoustic vortex to collect microbubbles noninvasively to increase the local concentration in vivo for drug residence, as shown in Figure 10a,b, a method that enables systemic drug delivery at very low doses. In the study of 3D acoustic tweezer biomanipulation, Ye Yang et al. [90] successfully achieved the manipulation of PDMS particles through a human skull based on a 2D planar array and combined with ultrasound imaging, time inversion algorithm, and acoustic tweezers. Figure 10c shows the distribution of the acoustic field in 3D space, the schematic diagram of particle motion trajectory, and the images after actual manipulation. This combination of multiple techniques makes acoustic manipulation in non-transparent and non-homogeneous media possible. Currently, achieving the manipulation of functional particles such as ultrasound contrast agents, cells, and bacteria in complex environments such as living bodies is an important development in research on the application of acoustic tweezer technology.

### 6.3. Applications in Micro-Robots

Micro-robots can access tissues or cavities that are difficult to reach by other devices in order to perform minimally invasive medical diagnostic or therapeutic operations. Amirreza Aghakhani et al. [91] proposed an acoustic wave-driven micro-robot that can effectively move through the human body at high speed in a minimally invasive manner under acoustically induced bubble oscillations. In addition, the microrobot can be combined with external magnetic forces to make it steerable [92], capable of driving and navigating in narrow body position regions and adjusting the acoustic wave amplitude allows for functions such as cargo capture, transport, and release of the microrobot. Using unfettered micro-robots in control of sub-millimeter-sized particles or payloads, Liqiang Ren et al. [93] proposed an acoustically driven bubble-based microswimmer, which is capable of autonomous motion in 3D space and can achieve precisely controllable motion as well as precise particle manipulation in 3D space. With further experimental research and theoretical development, the acoustic wave-based control micro-robot has promising applications in targeted drug delivery, detoxification, and non-invasive surgery.

### 6.4. Applications in Materials Science

In material preparation, acoustic manipulation can rely on acoustic radiation force against gravity to achieve the levitation of the target object in the acoustic radiation potential field, thus providing a container-free environment. Shen Changle et al. [94] used acoustic levitation to achieve the decomposition of calcium bicarbonate and the nucleation growth process of calcium carbonate, in which calcium carbonate is first precipitated as amorphous nuclei and then nuclei are formed in a suspended state on this basis, and this method can form single crystals of high quality [95]. In the study of alloy solidification, the application of acoustic manipulation can effectively avoid heterogeneous nucleation on the vessel wall and improve the cooling of the alloy melt; Yan Na et al. [96] effectively improved the cooling rate during the solidification of ternary Al-Cu-Si alloy by using acoustic streaming under the conditions of combined acoustic suspension and strong laser heating and prepared a finer organization and more uniform and dense alloy material. With technological progress, acoustic levitation-based technology has a broad application prospect in high-purity material processing, crystal growth, and pharmaceutical preparation purification.

## 7. Summary and Outlook

Currently, ultrasonic transducer array-based particle manipulation techniques have been increasingly used for the manipulation of cells, particles, and organisms, but most of the literature has focused on in vitro applications. Due to the non-invasive nature of acoustic waves and deep tissue penetration properties, ultrasonic particle manipulation has every potential to manipulate cells or foreign bodies in vivo, but less three-dimensional particle manipulation based on transducer arrays in living bodies in water has been reported. Because the difficulties in water compared with air are the following: (1) the electrical conductivity of water puts high requirements on array design and manufacturing process, and (2) the manipulation in water is not negligible by acoustic streaming factors. The challenges to realizing ultrasonic particle manipulation in vivo are the following: (1) non-transparent tissue structure hinders the visualization of particle manipulation based on optical images, and (2) acoustic distortion caused by non-uniform tissue poses a challenge to precise particle manipulation. To address these difficulties, the authors suggest that experimental studies on the 3D manipulation of particles such as cells in vivo should be carried out after solving the 3D manipulation of objects with different acoustic properties in water.

Ultrasonic particle manipulation, as a technology with practical application prospects, still has a large number of technical limitations to be solved. The authors believe that the future development direction of ultrasonic particle manipulation is in terms of hardware enhancement, such as (1) combining transducer arrays with artificial acoustic structures to achieve high-precision acoustic field dynamic modulation and precise manipulation beyond diffraction limits; (2) integrating with existing biomedical research systems and instruments and combining with image-guided technology to achieve in vivo visualization and precise manipulation in terms of application expansion, such as (1) combining with other non-contact manipulation means, such as acoustic tweezers and optical tweezers, magnetic tweezers, dielectric electrophoresis, etc., to realize manipulation under multi-physical field coupling.

In short, with the booming development of precision manufacturing and precision medicine, the precise manipulation of tiny particles is the core technology for the development of these fields. As a non-contact, non-damaging, particle manipulation technology that can achieve a variety of manipulation actions, acoustic manipulation can be miniaturized, integrated, and used intelligently for various devices and equipment. Further research on the theory of ultrasonic particle manipulation and the development of precise and sensitive acoustic manipulation technology for application scenarios is needed to provide core technology support for the realization of precision manufacturing and precision medicine.

## Figures and Tables

**Table 1:** Optimization methods and optimization of sound field reconstruction algorithms.

Algorithm	Control Method	Optimization Methods
GS-PTA	Amplitude and Phase	Improved GS algorithm
IBP/IASA	Phase	Improved GS algorithm
LMA	Phase	Damping least squares
Tikhonov	Amplitude and Phase	Regularization methods
Diff-PTA	Phase	Gradient drop

## Data Availability

No new data created.
